# Association of Albuminuria and Regression of Chronic Kidney Disease in Adults With Newly Diagnosed Moderate to Severe Chronic Kidney Disease

**DOI:** 10.1001/jamanetworkopen.2022.25821

**Published:** 2022-08-09

**Authors:** Meghann Pasternak, Ping Liu, Robert Quinn, Meghan Elliott, Tyrone Gorden Harrison, Brenda Hemmelgarn, Ngan Lam, Paul Ronksley, Marcello Tonelli, Pietro Ravani

**Affiliations:** 1 Departments of Medicine and Community Health Sciences, Cumming School of Medicine, University of Calgary, Calgary, Alberta, Canada

## Abstract

**Importance:**

People with chronic kidney disease (CKD) are risk-stratified for adverse events based on estimated glomerular filtration rate (eGFR) and albuminuria level. CKD has often a favorable course (CKD regression) regardless of eGFR. Determining whether lower albuminuria is associated with CKD regression may have implications on CKD management.

**Objective:**

To assess the 5-year probability of CKD regression across albuminuria categories accounting for the competing risks of CKD progression and death in people with newly diagnosed CKD and the association between albuminuria level and CKD regression.

**Design, Setting, and Participants:**

This population-based cohort study used administrative and laboratory data from Alberta, Canada, for adults with incident moderate to severe CKD (defined as sustained eGFR of 15-44 mL/min/1.73 m^2^ for >90 days), between April 1, 2008, and March 31, 2017, and albuminuria measures before cohort entry. Data analysis occurred in January to June 2022.

**Exposure:**

Albuminuria categories were defined by albumin to creatinine ratios (ACRs): A1 (ACR, <3 mg/mmol), A2 (ACR, 3-29 mg/mmol), A3_<60_ (ACR, 30-59 mg/mmol), and A3_≥60_ (ACR, ≥60 mg/mmol).

**Main Outcomes and Measures:**

The main outcome was time to the earliest of CKD regression or progression (sustained change in CKD stage for >3 months and ≥25% increase or decrease in eGFR from baseline or kidney failure, respectively), death, or censoring (outmigration or study end date: March 31, 2019). Cumulative incidence functions were used to estimate absolute risks, and cause-specific Cox models were used to assess the association between albuminuria and CKD regression, accounting for age, sex, eGFR, comorbidities, and health services use indicators.

**Results:**

Of 58 004 people with moderate to severe CKD (mean [SD] age, 77 [12] years; 31 725 [55%] women), 35 360 had A1 albuminuria (61%), 15 597 had A2 albuminuria (27%), 1527 had A3_<60_ albuminuria (3%), and 5520 had A3_≥60_ albuminuria (10%). Five-year probability of regression was highest with A1 (22.6%), followed by A2 (16.5%), and A3_<60_ (11.6%) and lowest with A3_≥60_ (5.3%). Using A1 albuminuria as the reference group, the hazard of regression was highest for A2 (hazard ratio [HR], 0.75; 95% CI, 0.72-0.79), then A3_<60_ (HR, 0.47; 95% CI, 0.40-0.54), and lowest for A3_≥60_ (HR, 0.27; 95% CI, 0.24-0.30).

**Conclusions and Relevance:**

In this cohort study of people with moderate to severe CKD, albuminuria levels were inversely associated with CKD regression. These findings extend the key prognostic role of albuminuria, offering novel opportunities to discuss both favorable and adverse outcomes in people with CKD and inform CKD management.

## Key Points

**Question:**

What is the association of albuminuria with chronic kidney disease (CKD) regression?

**Findings:**

In this cohort study of 58 004 adults with newly diagnosed moderate to severe CKD (eGFR, 15-44 mL/min/1.73 m^2^), albuminuria level was directly associated with CKD progression and death, and inversely associated with sustained improvement of eGFR for 90 days or longer. Five-year probabilities of CKD regression were higher in people with lower urine albumin-creatinine ratios in a stepwise fashion.

**Meaning:**

These findings suggest that there may be novel opportunities to discuss both favorable and adverse outcomes with adults with CKD.

## Teaser

This cohort study assesses the association of albuminuria levels with 5-year probability of chronic kidney disease (CKD) regression in adults with newly diagnosed CKD.

## Introduction

The higher prevalence of chronic kidney disease (CKD) with global population aging^1^ has raised concern over the potential increase in demand for health care services, including specialist nephrology care. Albuminuria level, a marker of kidney damage, and estimated glomerular filtration rate (eGFR), a marker of kidney function, are routinely used to assess the severity of CKD.^2,3^ Both eGFR and albuminuria have been informative in constructing risk-stratification strategies to tailor CKD management and guide appropriate resource allocation, including expedited referral of patients at higher risk of CKD progression.^3^

While recent evidence suggests that CKD regression (ie, sustained improvement in eGFR) occurs as often as progression,^4,5^ the traditional framing of CKD as an unrelenting and progressive process persists. Viewing CKD trajectory as a 1-way path toward failure may lead to an overly pessimistic prognosis outlook for patients and health care practitioners, hindering opportunities to improve health, and result in suboptimal CKD management. Studies have found that patients are often referred to nephrology despite not meeting regional criteria,^6,7,8^ possibly reflecting the belief among primary care physicians that progressive disease is inevitable. Although eGFR alone may not reliably differentiate individuals whose CKD progresses from those in whom CKD remains stable or regresses,^4,5,9,10^ referral is usually triggered by reduced eGFR, even in the absence of albuminuria, while referral criteria for albuminuria vary across guidelines.^11,12^ Finally, discharge back to primary care following referral is uncommon, despite the uncertain benefit of long-term nephrology care for many patients.^13^ Determining whether the probability of regression varies by degree of albuminuria could offer guidance to optimize CKD management and allow for more comprehensive patient education regarding CKD prognosis.

We performed a population-based study of adults with incident moderate to severe CKD to evaluate the 5-year probabilities of CKD regression, CKD progression, and death across clinically relevant categories of albuminuria, considering the recommended referral threshold of albuminuria. We also studied the independent association between albuminuria and CKD regression accounting for several baseline characteristics.

## Methods

This cohort study was approved by the institutional review boards at the Universities of Alberta and Calgary with a waiver of participant consent because of the retrospective design and secondary use of routinely collected administrative data. This study is reported following the Reporting of Studies Conducted Using Observational Routinely-Collected Data (RECORD) guidelines.

### Study Design and Data Sources

We used linked administrative and laboratory data from Alberta, Canada, for this population-based cohort study.^14^ Provincial population coverage of the database was complete since 2005 (eFigure 1 in the Supplement).

### Cohort Formation and Variable Definitions

We identified adults (aged ≥18 years) with newly documented sustained reduction of eGFR to 15 to 44 mL/min/1.73 m^2^ (CKD stages G4-G3b), between April 1, 2008, and March 31, 2017. We did not include adults with G3a CKD whose very low 5-year risk of kidney failure has implications on CKD definition, referral policies, and patient care.^4,15^ We calculated eGFR using the Chronic Kidney Disease Epidemiology Collaboration (CKD-EPI) 2009 equation, excluding the race coefficient, with serum creatinine values standardized to isotope dilution mass spectrometry–traceable methods.^16^ We used only outpatient eGFR measurements to minimize the inclusion of people with episodes of acute kidney injury. We used the mean value of eGFR when there were multiple measurements on the same day. To identify people with sustained reduction of eGFR, we screened each individual’s consecutive series of at least 2 eGFR measurements, where the first and last eGFR were separated by more than 90 days, the first and all possible intervening measurements within 90 days were 45 mL/min/1.73 m^2^ or less, and the last measure was 15 to 44 mL/min/1.73 m^2^. We selected the earliest series (qualifying period) where all eGFR measurements met the eGFR requirement for cohort entry (eTable 1 in the Supplement). The date of the last eGFR measurement in the qualifying period defined the index date (cohort entry date). We excluded individuals who had received kidney replacement therapy (chronic dialysis or kidney transplant) or with stage 5 CKD on or before the index date. To be eligible for inclusion, patients required at least 1 outpatient measurement of albuminuria within 3 years prior to the index date, with the following types of measurement in descending order of preference: albumin-to-creatinine ratio (ACR), protein-to-creatinine ratio (PCR), and urine dipstick protein.^12^ Patients were then identified as having sustained or nonsustained albuminuria levels, with sustained albuminuria defined as having 2 or more measures more than 90 days apart. Baseline albuminuria was the last measure of albuminuria obtained prior to the index date. We converted urine dipstick and PCR measures to ACR using a validated conversion formula.^17^ We classified patients into 4 ACR categories: A1 (ACR, <3 mg/mmol), A2 (ACR, 3-29 mg/mmol), A3_<60_ (ACR, 30-59 mg/mmol), and A3_≥60_ (ACR, ≥60 mg/mmol). We used validated algorithms to identify comorbidities.^18^

### Outcomes

We studied time to the first of CKD regression (primary outcome), CKD progression, or death. *Regression* was defined as an increase in eGFR of 25% or more from baseline and sustained improvement of CKD stage, using 2 measures separated by more than 90 days (eTable 1 in the Supplement). *Progression* was defined as a decrease in eGFR of 25% or more from baseline and a worsening of CKD stage, using 2 measures separated by more than 90 days. Kidney failure, defined as the earliest of initiation of chronic kidney replacement therapy (obtained from provincial registry data), was included among the outcome of progression. We censored observations at study end (March 31, 2019) or emigration from province.

### Statistical Analysis

We used the Aalen-Johnson method^19^ for estimating the cumulative incidence functions of each competing event overall and across albuminuria categories. We further stratified these analyses by age and eGFR stage and summarized absolute risks at 5 years using bar-plots. We evaluated the associations between albuminuria and CKD regression using cause-specific Cox regression,^20^ accounting for age, sex, eGFR, comorbidities (ie, diabetes, coronary artery disease, peripheral vascular disease, congestive heart failure, and stroke), and markers of access to health services (number of eGFR measurements during the qualifying period and dispensed medications including statins, angiotensin pathway inhibitors, and nonsteroidal antiinflammatory drugs). The association between albuminuria and CKD regression was estimated assuming absence and presence of effect modification by CKD severity (eGFR categories defined by 5-mL/min/1.73 m^2^ increments) and considering possible additional interactions (eg, between age and eGFR). We used residual analyses to identify deviations from the linearity and proportionality assumption. During model building, we checked that results were consistent across study time.

We checked consistency of results redefining CKD progression as incident chronic kidney replacement therapy only, instead of CKD progression or kidney replacement, and across subgroups defined by (1) diabetes status, (2) measured vs calculated ACR, (3) timing of albuminuria measurement (time 0 and 0-1, 1-2, and 2-3 years before study entry), (4) outpatient eGFR documentation before the qualifying period; or (5) sustained vs nonsustained albuminuria. Since we expected few people with A1 albuminuria would have repeated measures, we included nonsustained A1 albuminuria as the reference category in Cox regression of the fifth subgroup analysis. We summarized the direct association between albuminuria and the cumulative incidence of CKD regression using Fine and Gray regression.^21^ We conducted analyses in January to June 2022. Statistical analyses were conducted using R software version 4.2.0 (R Project for Statistical Computing). *P* values were 2-sided, and statistical significance was set at *P* = .05.

## Results

### Study Cohort

We identified 65 509 adults with newly documented G3b or G4 CKD during the study period (eTable 2 and eFigure 1 in the Supplement). Of these, 7505 individuals (11.5%) had no recorded measures of albuminuria within the 3-year prior to index date. The proportion of people without albuminuria measurements before G3b to G4 CKD documentation remained stable across the study period (eFigure 2 in the Supplement). The final study cohort included 58 004 individuals (mean [SD] age, 77 [12] years; 31 725 [55%] women). Compared with the people who had measurements of albuminuria and were included in the final study cohort, excluded individuals were more likely to be classified as stage G3b and to be women and less likely to have diabetes (eTable 2 in the Supplement). Of these individuals, only 939 (13%) had a measure of albuminuria by 3 months of follow-up.

In the final study cohort, 35 360 participants (61%) had A1 albuminuria, followed by 15 597 participants with A2 albuminuria (27%), 1527 participants with A3_<60_ albuminuria (3%), and 5520 participants with A3_≥60_ albuminuria (10%) (Table). People with A3_<60_ and A3_≥60_ albuminuria were younger, more likely to be men, and more likely to have diabetes. Most study participants had at least 1 outpatient eGFR measurement of 45 mL/min/1.73 m^2^ or greater recorded before study entry (55363 participants [96%]).

**Table.** Baseline Characteristics by Category of Albuminuria^a^

Characteristics	Individuals, No. (%)				
	All (N = 58 004)	A1 (n = 35 360)	A2 (n = 15 597)	A3_<60_ (n = 1527)	A3_≥60_ (n = 5520)
Age, y					
Median (IQR)	78 (70-85)	80 (73-86)	78 (69-85)	72 (62-80)	67 (56-77)
<70	14 383 (25)	6366 (18)	4104 (26)	668 (44)	3245 (59)
70-79	18 109 (31)	11 374 (32)	4944 (32)	474 (31)	1317 (24)
80-84	11 219 (19)	7564 (21)	2891 (19)	220 (14)	544 (10)
≥85	14 293 (25)	10 056 (28)	3658 (23)	165 (11)	414 (8)
Sex					
Women	31 725 (55)	21 822 (62)	7283 (47)	565 (37)	2055 (37)
Men	26 279 (45)	13 538 (38)	8314 (53)	962 (63)	3465 (63)
Qualifying period, median (IQR), d	168 (112-292)	182 (118-329)	154 (109-257)	139 (106-208)	132 (105-197)
eGFR tests during qualifying period, median (IQR), No.	2 (2-3)	2 (2-3)	2 (2-3)	3 (2-4)	3 (2-4)
Outpatient eGFR before qualifying period					
No prior eGFR	2641 (5)	1550 (4)	693 (4)	83 (5)	315 (5)
Prior eGFR recorded	55 363 (95)	33 810 (96)	14 904 (96)	1444 (95)	5205 (94)
Index eGFR, median (IQR), mL/min/1.73 m^2^	38 (33-42)	39 (35-42)	37 (31-41)	36 (29-41)	34 (26-39)
CKD stage					
G3b	48 376 (83)	31 320 (89)	12 383 (79)	1094 (72)	3579 (65)
G4	9628 (17)	4040 (11)	3214 (21)	433 (28)	1941 (35)
Albuminuria measure					
ACR	27 052 (47)	13 387 (38)	8716 (56)	1193 (78)	3756 (68)
Dipstick protein	27 033 (47)	20 759 (59)	5542 (36)	0	732 (13)
PCR	3919 (7)	1214 (3)	1339 (8)	334 (22)	1032 (19)
Sustained proteinuria	14 346 (25)	11 (<1)	8407 (54)	1238 (81)	4690 (85)
Comorbidities					
Cardiovascular disease	27 742 (48)	16 382 (46)	8078 (52)	724 (47)	2558 (46)
Myocardial infarction	5818 (10)	3304 (9)	1780 (11)	156 (10)	578 (10)
Congestive heart failure	16 770 (29)	9661 (27)	5085 (33)	444 (29)	1580 (29)
Peripheral vascular disease	3764 (7)	2037 (6)	1165 (8)	120 (8)	442 (8)
Stroke or TIA	13 218 (23)	7992 (23)	3723 (24)	304 (20)	1199 (22)
Diabetes	25 727 (44)	12 002 (34)	8664 (56)	1072 (70)	3989 (72)
Dispensed medications					
ACEI/ARB	44 622 (77)	26 616 (75)	12 090 (78)	1267 (83)	4649 (84)
NSAIDs	11 190 (19)	7371 (21)	2768 (18)	219 (14)	832 (15)
Statins	30 299 (52)	17 057 (48)	8712 (56)	963 (63)	3567 (65)

Abbreviations: ACEI, angiotensin-converting enzyme inhibitor; ACR, albumin to creatinine ratio; ARB, angiotensin-receptor blocker; eGFR, estimated glomerular filtration rate; NSAID, nonsteroidal anti-inflammatory drug; PCR, protein-creatinine ratio; TIA, transient ischemic attack.

SI conversion factor: To convert ACR from milligrams per gram, multiply by 0.113.

^a^ Categories of albuminuria were based on converted and unconverted ACR and defined as A1, less than 3 mg/mmol; A2, 3 to 29 mg/mmol; A3_<60_, 30 to 59 mg/mmol; and A3_≥60_, 60 mg/mmol or greater. Converted values were obtained using validated conversion method.^15^

### Absolute Risks

At 5 years from cohort entry, 14 545 participants (25%) had died, 10 299 participants (18%) had CKD regression, and 9266 participants (16%) had either progressed to stage G4 CKD (7211 participants [78%]) or G5-CKD (1521 participants [16%], of whom 545 participants [36%] started chronic kidney replacement therapy). Less than 1% of the study participants who were censored or event-free at the study end date did not have a recorded outpatient eGFR during follow-up. The probability of regression was higher than the probability of progression in people with or without albuminuria data, with the group without albuminuria data showing higher mortality (eFigure 3 in the Supplement).

**Figure 1.**
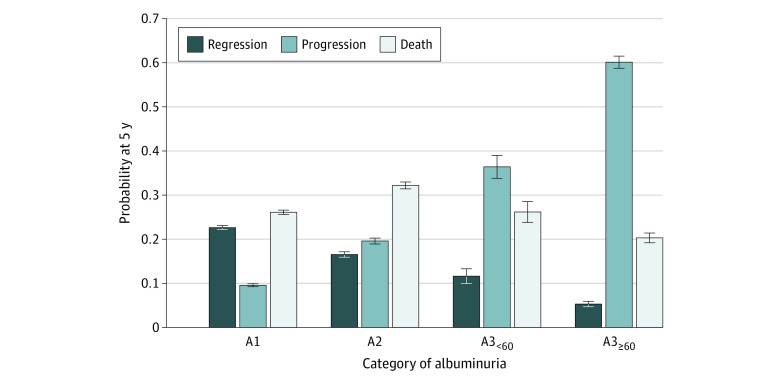
Outcome Probabilities at 5 Years From Study Entry by Category of Albuminuria Outcome probabilities were estimated using cumulative incidence functions at 5 years after study entry by category of albuminuria. Progression represents chronic kidney disease (CKD) progression or kidney failure. Death refers to death without regression, progression, or kidney failure. Albuminuria was categorized according to albumin to creatinine ratio, with A1 indicating less than 3 mg/mmol; A2, 3 to 30 mg/mmol; A3_<60_, 30 to 60 mg/mmol; and A3_≥60_ 60 mg/mmol or greater.

The probability of regression at 5 years decreased with increasing degree of albuminuria, from 22.6% for A1 to 16.5% for A2, 11.6% for A3_<60_, and 5.3% for A3_≥60_ (Figure 1). The risk of CKD progression or kidney failure had a proportionally larger and opposite trend (9.6% for A1, 19.6% for A2, 36.4% for A3_<60_, and 60.1% for A3_≥60_ ). The 5-year risk of death without kidney failure varied with a less evident trend across increased albuminuria category (26.1% for A1, 32.2% for A2, 26.1% for A3_<60_, and 20.3% for A3_≥60_).

**Figure 2.**
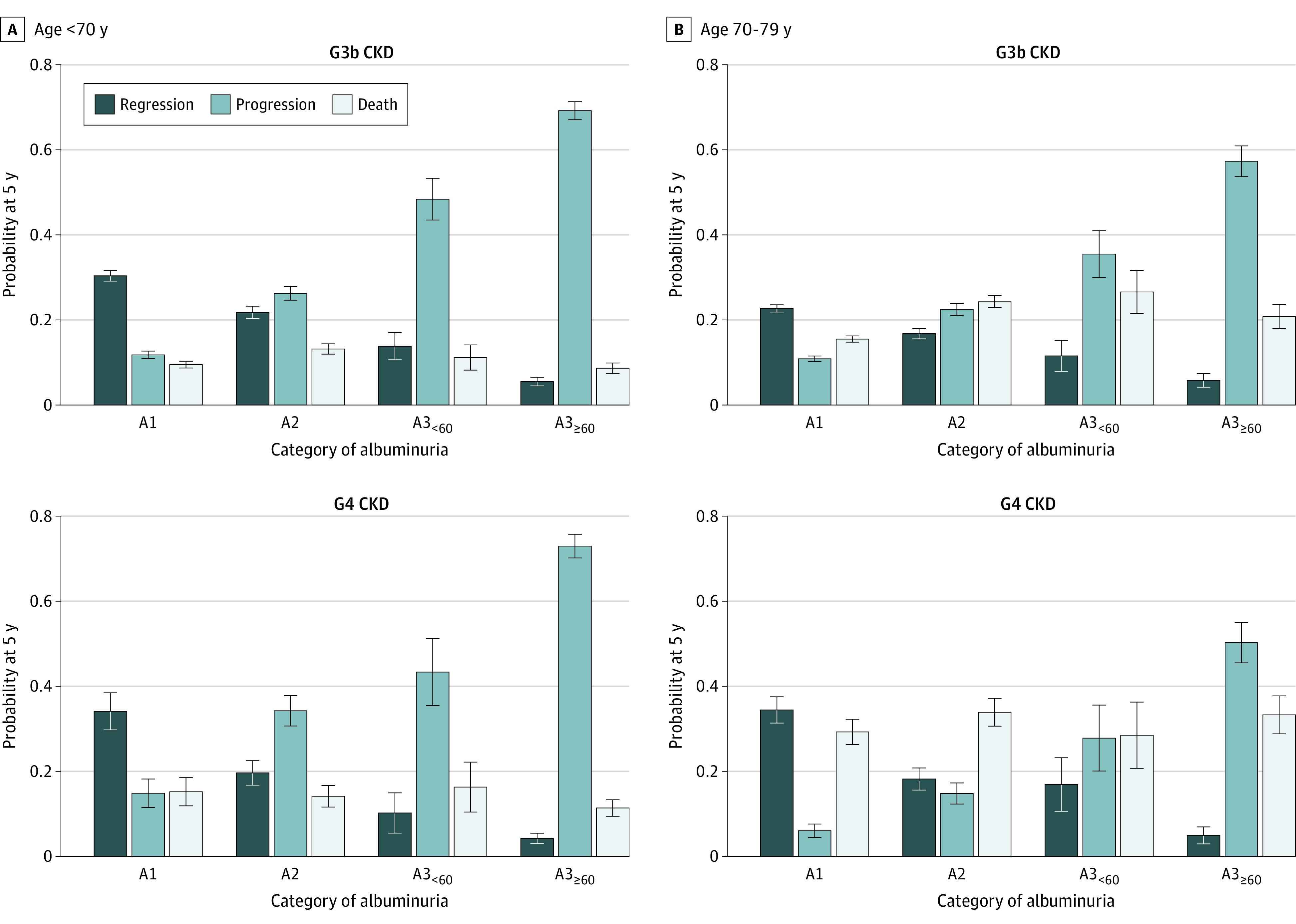
Outcome Probabilities at 5 Years From Study Entry by Category of Albuminuria Stratified by Estimated Glomerular Filtration Rate Among Patients Younger than 80 Years Outcome probabilities were estimated using cumulative incidence functions at 5 years after study entry by category of albuminuria, stratified by category of estimated glomerular filtration rate and age. Progression represents chronic kidney disease (CKD) progression or kidney failure. Death refers to death without regression, progression, or kidney failure. Albuminuria was categorized according to albumin to creatinine ratio, with A1 indicating less than 3 mg/mmol; A2, 3 to 30 mg/mmol; A3_<60_, 30 to 60 mg/mmol; and A3_≥60_ 60 mg/mmol or greater.

**Figure 3.**
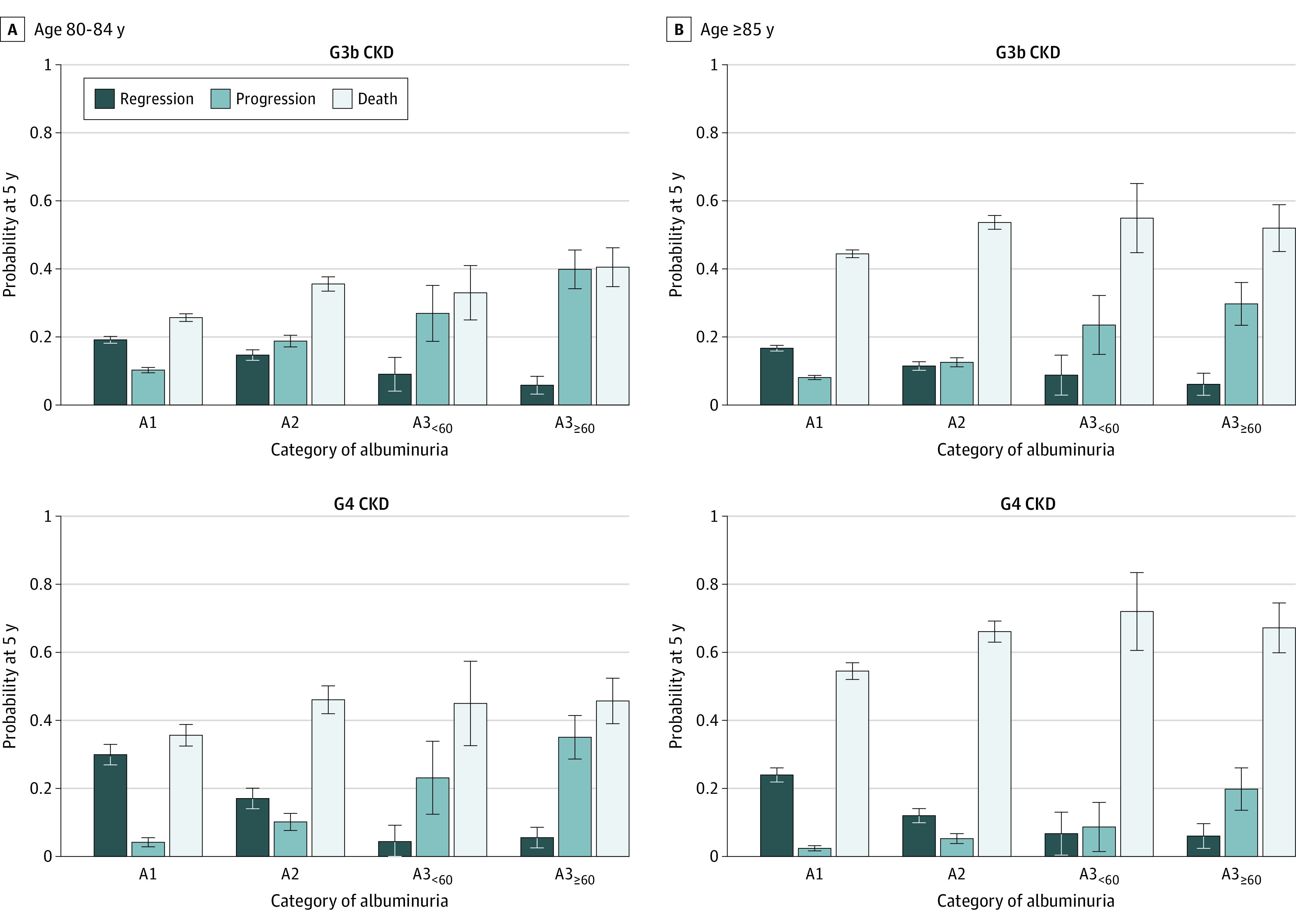
Outcome Probabilities at 5 Years From Study Entry by Category of Albuminuria Stratified by Estimated Glomerular Filtration Rate Among Patients Aged 80 Years and Older Outcome probabilities were estimated using cumulative incidence functions at 5 years after study entry by category of albuminuria, stratified by category of estimated glomerular filtration rate and age. Progression represents chronic kidney disease (CKD) progression or kidney failure. Death refers to death without regression, progression, or kidney failure. Albuminuria was categorized according to albumin to creatinine ratio, with A1 indicating less than 3 mg/mmol; A2, 3 to 30 mg/mmol; A3_<60_, 30 to 60 mg/mmol; and A3_≥60_ 60 mg/mmol or greater.

The probability of regression decreased with increasing age only with A1 and A2 albuminuria. People with A3_<60_ or A3_≥60_ had the lowest probability of regression, which remained stable across increasing age (Figure 2 and Figure 3). Patterns were similar across CKD stage categories G3b and G4.

### Measures of Association

**Figure 4.**
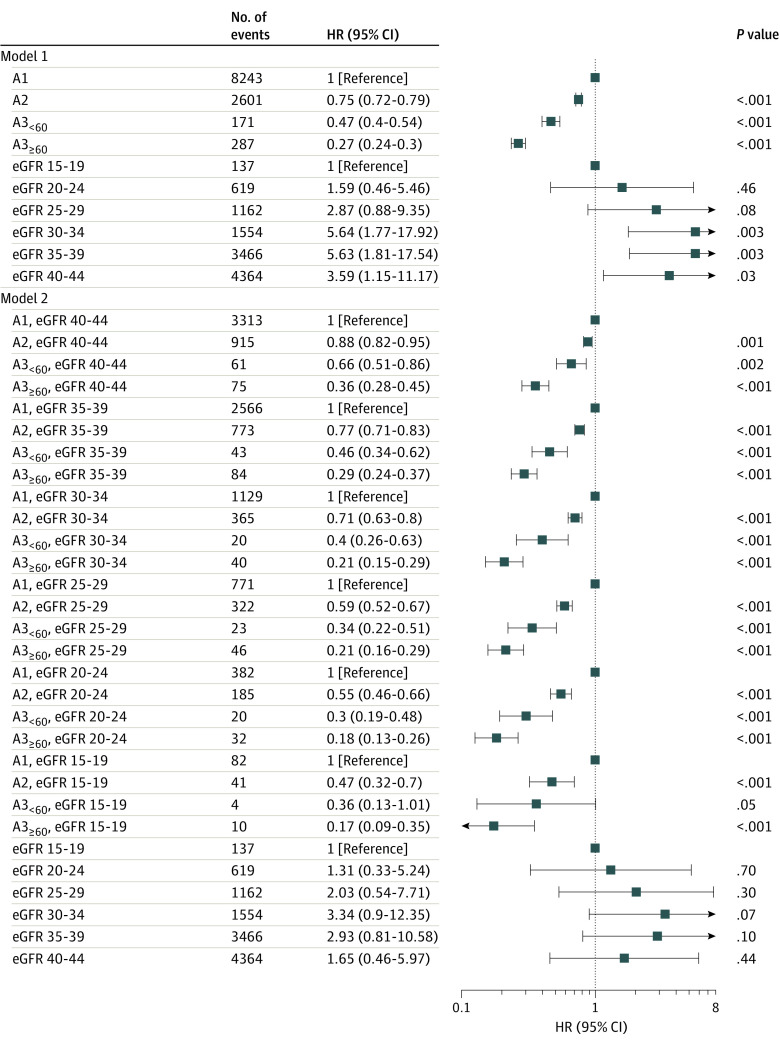
Association Between Albuminuria and Chronic Kidney Disease (CKD) Regression Model 1 does not include the interaction between albuminuria and estimated glomerular filtration rate (eGFR) (eTable 3 in the Supplement). Model 2 includes the same covariates as Model 1 with the additional interaction between albuminuria and index eGFR category (eTable 4 in the Supplement). Model 2 shows the linear combinations of the coefficients (epidemiological formulation) instead of differences in log-hazard ratios (HRs) (statistical interaction formulation; eTable 4 in the Supplement) to summarize the association between eGFR and CKD regression across categories of albuminuria. An alternative formulation of model 2 that summarizes the association between eGFR and CKD regression across categories of albuminuria is presented in eFigure 4 in the Supplement.

Higher levels of albuminuria were associated with significantly lower hazards of regression and higher hazards of CKD progression and death (eTable 3 in the Supplement; Figure 4). Compared with albuminuria that was within reference range to mild (A1), people with A3_≥60_ were least likely to experience regression (hazard ratio [HR], 0.27; 95% CI, 0.24-0.30), followed by A3_<60_ (HR, 0.47; 95% CI, 0.40-0.54) and A2 (HR, 0.75; 95% CI, 0.72-0.79). The pattern of association between albuminuria and regression did not change when we considered the effect modification by eGFR, although the association became stronger with lower eGFR (*P* for interaction <.001) (Figure 4; eFigure 4 and eTable 4 in the Supplement). According to this model, there was no clear trend in the association between eGFR and regression (eFigure 4 in the Supplement).

### Other Analyses

We found similar results when we redefined CKD progression as incident chronic kidney replacement therapy only instead of CKD progression or kidney replacement (eFigure 5 and eTable 5 in the Supplement) and in analyses of subgroups defined by diabetic status, measured vs calculated baseline albuminuria measurement, timing of albuminuria measurement, outpatient eGFR documentation before the qualifying period, and sustained albuminuria (eFigures 6-10 and eTable 5 in the Supplement). eTable 6 in the Supplement summarizes the results of the cause-specific Cox and Fine and Gray models.

## Discussion

In this population-based cohort study, we found a significant and inverse association between albuminuria and CKD regression in patients with incident moderate to severe CKD. First, increasing levels of albuminuria were associated with an incremental decrease in the probability of regression during follow-up, independent of eGFR and age. Compared with patients with A1 albuminuria, regression of CKD was less likely to occur in those with A2, A3_<60_, or A3_≥60_ albuminuria. Second, more than half of the study population had A1 albuminuria. Within this large group, regression was more likely than progression or kidney failure regardless of baseline eGFR or age, and participants in the younger proportion of this group (age <80 years) were more likely to regress than die. These findings support the importance of albuminuria in delineating CKD outcomes and suggest that degree of albuminuria is not only a mutual risk factor of progression along with eGFR, but also a strong and unique indicator of CKD regression.

Many studies have demonstrated that increasing albuminuria, a reliable marker of kidney damage, is associated with higher rates of CKD progression or kidney failure.^2,3,22,23^ According to our findings, increasing albuminuria was also associated with significant decrease in the rates of CKD regression, independent of eGFR. Conversely, the association between eGFR levels and CKD regression is uncertain.^4,5,9^ While regression can occur at any degree of kidney function impairment, the probability of improvement may largely depend on the extent of kidney damage, as indicated by the degree of albuminuria.

Our study has health policy and clinical implications. Given the high demand for referral to specialist nephrology care relative to available resources,^7,13^ timing of referral and the decision to discharge back to primary care should consider both the absolute risk of CKD progression and the probability of CKD regression. While probabilities of regression and progression are comparable across CKD stages,^4,5^ albuminuria measures could help identify patients with more stable disease from those who are more or less likely to improve. In the setting of albuminuria that is within reference range to mild, further follow-up at the primary care level may be advised, since regression is common, especially in moderate G3b CKD. On the other hand, with higher albuminuria, regression becomes improbable and expedited referral for specialist care may be warranted. Although there is consensus that nephrology referral should occur once eGFR is less than 30 mL/min/1.73 m^2^ (stage G4 CKD), guidelines differ regarding referral recommendations based on ACR.^8,11,12^ We found that compared with people with G3b CKD with ACR less than 3 mg/mmol (54% of the cohort), those with G3b CKD and ACR of 30 to 59 mg/mmol CKD (2% of the cohort) had 44% to 60% lower hazard rates of regression. This small subset of patients with eGFR greater than 30 mL/min/1.73 m^2^ (stage G3b CKD) and ACR 30 to 59 mg/mmol may benefit from earlier nephrology referral, as recommended by some (but not all) guidelines.^8,11,12^ Although the probability of regression is reduced to a lesser extent in patients with stage G3b CKD and ACR of 3 to 29 mg/mmol (12%-29% lower hazard), their increased absolute risk of progression relative to those without albuminuria requires closer monitoring by their primary care physicians. Since patients with albuminuria that is mild or within reference range and G3b CKD comprise the largest proportion of the CKD population, these findings could result in a significant reduction of potentially inappropriate referrals, granting access to those most likely to benefit from consultation.

Finally, patients’ perception of a disease as a ceaseless and progressive process can have deleterious effects on mental health and clinical outcomes.^24,25^ Compared with individuals with undiagnosed hypertension, people with a known diagnosis of hypertension report lower health-related quality of life, independent of symptoms.^23,26^ Studies also suggest that compared with optimism, pessimism in chronic disease is associated with reduced self-efficacy and poor adherence to recommended health behaviors.^27,28^ The unrelenting nature of chronic illness may evoke a sense of learned helplessness due to a perceived lack of control over the clinical outcome. Furthermore, patient education in CKD may slow progression and delay time to kidney replacement therapy.^29,30^ Our findings suggest that clinicians can empower their patients by providing a more optimistic outlook on disease prognosis, allowing patients and health care practitioners to better engage in shared decision-making and positively impact the psychosocial aspects of health.

Although albuminuria is possibly one of the most important factors associated with CKD outcomes, it is not consistently measured in people with CKD. First, in our study, approximately 10% of people with sustained eGFR reduction consistent with moderate to severe CKD had not been tested for albuminuria within 3 years prior to diagnosis, despite a quarter of these people having confirmed diabetes. This pattern has not changed in the last decade, and of the individuals without preceding measures of albuminuria, only 13% had albuminuria measured in 3 months following CKD diagnosis. These findings are complementary to recent work examining quality of CKD management in Canada that found that only 18.4% of people had follow-up albuminuria measures within 6 months of CKD diagnosis.^31^ Second, only 25% of people in our cohort had follow-up measures of proteinuria within the 3-year time frame preceding diagnosis of moderate to severe CKD. Repeated albuminuria measures were least common in those with A1 albuminuria (<1% of patients) despite more than one-third of this group being diagnosed with diabetes. Although higher mortality in people without albuminuria measurements may reflect selection bias (ie, people who are more severely ill are less likely to be tested), these findings suggest a need to address a possible lack of awareness regarding the importance of albuminuria in the screening and management of CKD.

Our study has implications for future research. First, the pathologic relationship between albuminuria and CKD outcomes is not fully understood. Existing evidence suggests that persistent exposure to albumin at the level of the glomerulus leads to potentially irreversible fibrotic changes.^32^ Although this may explain why regression is less likely with more severe albuminuria, we do not know what structural changes may occur in CKD regression. Clarification of the mechanistic basis of regression could identify potentially modifiable factors that could be targeted by interventions. Also, our study examined the association between baseline albuminuria and probability of regression and did not assess outcomes after regression or the impact of subsequent changes in albuminuria. Although pharmacologic reduction in albuminuria is known to slow the rate of disease progression,^33,34,35^ the impact of these interventions on CKD regression are unclear.^33^

This study is novel and has strengths. Previous studies have shown that CKD regression and death are more likely than CKD progression or kidney failure with advancing age,^4^ and that CKD regression is the most common outcome in primary care settings.^4,5,9,10^ To our knowledge, the interplay between eGFR and albuminuria on the probability of regression has never been assessed using a large sample size of patients drawn from a population with universal access to health care. Also, we applied a chronicity criterion to define CKD following guideline recommendations.^12^ Finally, we evaluated albuminuria using clinically relevant categories allowing for application to clinical decision-making.

### Limitations

Our study should be interpreted in the context of its potential limitations. First, it is possible that CKD regression, as we defined it, may have been influenced by regression-to-the-mean^36^ or kidney function recovery from an episode of acute kidney injury. Although we used 2 or more eGFR outpatient measures more than 90 days apart to define regression^12^ we cannot exclude that outcome misclassification might have occurred. Second, weight loss or malnutrition is an alternative explanation for increasing eGFR. Although some studies that evaluated group differences between regressors and nonregressors have not found a significant difference in body mass index or serum albumin levels,^9,10^ we could not include weight change as a potential confounder. Third, given its observational design, we could only account for measured confounders; thus, causal inference cannot be drawn from these data. More favorable outcomes in people with lower albuminuria may be due to individual characteristics, social determinants of health, or better access to care. Additionally, patient cohorts were formed using albuminuria categories that required the conversion of PCR and urine dipstick measures into ACR. Although this approach may have led to misclassification of some individuals, most study participants had direct measures of ACR, we used validated conversion methods, and albuminuria categories had a broad range; therefore, the impact of minor conversion errors was limited. Analyses restricted to participants with measured ACR, or sustained albuminuria were consistent with results of main analyses.

## Conclusions

This cohort study found that albuminuria was associated with CKD regression in patients with incident moderate to severe CKD. The degree of albuminuria could be used to inform referral practices by incorporating probability of CKD progression and regression. Furthermore, evaluating CKD with albuminuria could help clinicians and patients engage in a more comprehensive discussion that includes both adverse and favorable outcomes and positively impact the psychosocial aspects of health for people with CKD.

## Notes

Supplement. **eTable 1.** Sustained eGFR Methods for Cohort Formation and Kidney Outcome Ascertainment **eTable 2.** Baseline Characteristics Including Missing Values of Proteinuria **eTable 3.** Cause-Specific Hazard Ratios for Outcomes (No Interaction Model) **eTable 4.** Cause-Specific Hazard Ratios for Outcomes (Interaction Model) **eTable 5.** Cause-Specific Hazard Ratios for Regression (Sensitivity and Subgroup Analyses) Associated With Albuminuria **eTable 6.** Cause-Specific Hazard Ratios, Subdistribution Hazard Ratios for Regression Associated With Albuminuria **eFigure 1.** Derivation of Study Cohort **eFigure 2.** Study Participants With Missing Albuminuria Measures Per Year Prior to the Study Period **eFigure 3.** Cumulative Incidence Functions for Outcomes in People With Complete and Missing Albuminuria Data **eFigure 4.** Association Between eGFR and CKD Regression Across Albuminuria Categories **eFigure 5.** Crude 5-Year Risk of Outcomes by Category of Albuminuria (Sensitivity Analysis) **eFigure 6.** Crude 5-Year Risk of Outcomes by Diabetes Status **eFigure 7.** Crude 5-Year Risk of Outcomes According to the Type of Albuminuria Measurement **eFigure 8.** Crude 5-Year Risk of Outcomes According to Timing of Albuminuria Measurement **eFigure 9.** Crude 5-Year Risk of Outcomes in Participants Without and With eGFR Before Study Entry **eFigure 10.** Crude 5-Year Risk of Outcomes in Participants With and Without Sustained Albuminuria at Baseline
