# Supplementary material for: Association of Albuminuria and Regression of Chronic Kidney Disease in Adults With Newly Diagnosed Moderate to Severe Chronic Kidney Disease
Source: JAMA Netw Open. 2022 Aug 9;5(8):e2225821. doi: 10.1001/jamanetworkopen.2022.25821 (PMC9364131; doi:10.1001/jamanetworkopen.2022.25821)
Supplement: Supplement. — eTable 1. Sustained eGFR Methods for Cohort Formation and Kidney Outcome Ascertainment eTable 2. Baseline Characteristics Including Missing Values of Proteinuria eTable 3. Cause-Specific Hazard Ratios for Outcomes (No Interaction Model) eTable 4. Cause-Specific Hazard Ratios for Outcomes (Interaction Model) eTable 5. Cause-Specific Hazard Ratios for Regression (Sensitivity and Subgroup Analyses) Associated With Albuminuria eTable 6. Cause-Specific Hazard Ratios, Subdistribution Hazard Ratios for Regression Associated With Albuminuria eFigure 1. Derivation of Study Cohort eFigure 2. Study Participants With Missing Albuminuria Measures Per Year Prior to the Study Period eFigure 3. Cumulative Incidence Functions for Outcomes in People With Complete and Missing Albuminuria Data eFigure 4. Association Between eGFR and CKD Regression Across Albuminuria Categories eFigure 5. Crude 5-Year Risk of Outcomes by Category of Albuminuria (Sensitivity Analysis) eFigure 6. Crude 5-Year Risk of Outcomes by Diabetes Status eFigure 7. Crude 5-Year Risk of Outcomes According to the Type of Albuminuria Measurement eFigure 8. Crude 5-Year Risk of Outcomes According to Timing of Albuminuria Measurement eFigure 9. Crude 5-Year Risk of Outcomes in Participants Without and With eGFR Before Study Entry eFigure 10. Crude 5-Year Risk of Outcomes in Participants With and Without Sustained Albuminuria at Baseline [file jamanetwopen-e2225821-s001.pdf]

Supplemental Online Content

Pasternak M, Liu P, Quinn R, et al. Association of albuminuria and regression of chronic kidney disease in adults with newly diagnosed moderate to severe chronic kidney disease. *JAMA Netw Open*. 2022;5(8):e2225821. doi:10.1001/jamanetworkopen.2022.25821

eTable 1. Sustained eGFR Methods for Cohort Formation and Kidney Outcome Ascertainment
eTable 2. Baseline Characteristics Including Missing Values of Proteinuria
eTable 3. Cause-Specific Hazard Ratios for Outcomes (No Interaction Model)
eTable 4. Cause-Specific Hazard Ratios for Outcomes (Interaction Model)
eTable 5. Cause-Specific Hazard Ratios for Regression (Sensitivity and Subgroup Analyses) Associated With Albuminuria
eTable 6. Cause-Specific Hazard Ratios, Subdistribution Hazard Ratios for Regression Associated With Albuminuria
eFigure 1. Derivation of Study Cohort
eFigure 2. Study Participants With Missing Albuminuria Measures Per Year Prior to the Study Period
eFigure 3. Cumulative Incidence Functions for Outcomes in People With Complete and Missing Albuminuria Data
eFigure 4. Association Between eGFR and CKD Regression Across Albuminuria Categories
eFigure 5. Crude 5-Year Risk of Outcomes by Category of Albuminuria (Sensitivity Analysis)
eFigure 6. Crude 5-Year Risk of Outcomes by Diabetes Status
eFigure 7. Crude 5-Year Risk of Outcomes According to the Type of Albuminuria Measurement
eFigure 8. Crude 5-Year Risk of Outcomes According to Timing of Albuminuria Measurement
eFigure 9. Crude 5-Year Risk of Outcomes in Participants Without and With eGFR Before Study Entry
eFigure 10. Crude 5-Year Risk of Outcomes in Participants With and Without Sustained Albuminuria at Baseline

This supplemental material has been provided by the authors to give readers additional information about their work.

© 2022 Pasternak M et al. *JAMA Network Open.*

eTable 1. Sustained eGFR Methods for Cohort Formation and Kidney Outcome Ascertainment

**Cohorts**	**Qualifying period for entry**				**Outcomes**	**Qualifying period for outcomes**		
	First eGFR	*Possible intervening measurements within 90-days of the first*	*Last eGFR (Index date) >90 days from first*			*First eGFR*	*Possible intervening measurements within 90-days of the first*	*Last eGFR (outcome date) >90 days from first*
Moderate CKD	<45	<45	≥30 to <45		Regression	≥45	≥45	≥45
					Progression	<30	<30	<30
					Kidney $failure_{eGFR}$	<15	<15	<15
Severe CKD	<30	<30	≥15 to <30		Regression	≥30	≥30	≥30
					Kidney $failure_{eGFR}$	<15	<15	<15

Legend: CKD regression was defined as a sustained improvement to a better eGFR category for >3 months (>45 for G3b or >30 for G4), accompanied by a 25% or greater increase in the last eGFR from baseline (eGFR at index date). CKD progression was defined as a sustained decrease in eGFR below 30 for CKD stage G3b or below 15 for G4) for >3 months, accompanied by a 25% or greater drop in the last eGFR from baseline (eGFR at index date).

© 2022 Pasternak M et al. *JAMA Network Open.*

eTable 2. Baseline Characteristics Including Missing Values of Proteinuria

**Characteristics**	**All**	**Measured**	**Unmeasured**
	**N = 65,509**	N = 58,004	N = 7,505
Age (years)	79 (71, 85)	78 (70, 85)	83 (75, 89)
<70	15,455 (24)	14,383 (25)	1,072 (14)
70-79	19,897 (30)	18,109 (31)	1,788 (24)
80-84	12,654 (19)	11,219 (19)	1,435 (19)
≥85	17,503 (27)	14,293 (25)	3,210 (43)
Sex			
Female	36,300 (55%)	31,725 (55)	4,575 (61)
Male	29,209 (45%)	26,279 (45)	2,930 (39)
Qualifying period (QP, days)	171 (113, 305)	168 (112, 292)	211 (124, 407)
N of eGFR tests during	2 (2, 3)	2 (2, 3)	2 (2, 3)
Outpatient eGFR before QP			
No prior eGFR	3,497 (5.3%)	2,641 (5)	856 (11)
Prior eGFR recorded	62,012 (95%)	55,363 (95)	6,649 (89)
Index eGFR (mL/min/1.73 m^2)	38 (33, 42)	38 (33, 42)	38 (33, 42)
CKD stage			
G3b	54,725 (84%)	48,376 (83)	6,349 (85)
G4	10,784 (16%)	9,628 (17)	1,156 (15)
Comorbidities	32,002 (49%)		
Cardiovascular disease	6,685 (10%)	27,742 (48)	4,260 (57)
Myocardial infarction	19,617 (30%)	5,818 (10)	867 (12)
Congestive heart failure	4,239 (6.5%)	16,770 (29)	2,847 (38)
Peripheral vascular disease	15,204 (23%)	3,764 (7)	475 (6)
Stroke or TIA	27,524 (42%)	13,218 (23)	1,986 (26)
Diabetes	49,822 (76%)	25,727 (44)	1,797 (24)
Dispensed medications	12,485 (19%)		
ACEi/ARB	33,137 (51%)	44,622 (77)	5,200 (69)
NSAIDs	171 (113, 305)	11,190 (19)	1,295 (17)
Statins	2 (2, 3)	30,299 (52)	2,838 (38)

ACEi, angiotensin-converting enzyme inhibitor; ARB, angiotensin-receptor blocker; eGFR, estimated glomerular filtration rate; NSAIDs, non-steroidal anti-inflammatory drugs; PCR, protein-creatinine ratio; TIA, transient ischemic attack. Values are median (inter quartile range) or number (%).

© 2022 Pasternak M et al. *JAMA Network Open.*

eTable 3. Cause-Specific Hazard Ratios for Outcomes (No Interaction Model)

	**Hazard ratio (95% confidence interval)**		
**Covariates**	**Regression**	**Progr. or KF**	**Death**
Albuminuria[a]			
A1	1 (reference)	1 (reference)	1 (reference)
A2	0.75 (0.72, 0.79)	1.84 (1.75, 1.93)	1.31 (1.27, 1.36)
$A3_{<60}$	0.47 (0.40, 0.54)	2.92 (2.67, 3.20)	1.36 (1.23, 1.50)
$A3_{\geq 60}$	0.27 (0.24, 0.30)	5.64 (5.34, 5.97)	1.50 (1.40, 1.60)
Index eGFR[b]			
15-19	1 (reference)	1 (reference)	1 (reference)
20-24	1.59 (0.46, 5.46)	0.47 (0.31, 0.72)	0.76 (0.32, 1.78)
25-29	2.87 (0.88, 9.35)	0.21 (0.14, 0.32)	0.59 (0.26, 1.34)
30-34	5.64 (1.77, 17.92)	0.16 (0.11, 0.24)	0.56 (0.26, 1.22)
35-39	5.63 (1.81, 17.54)	0.09 (0.06, 0.13)	0.32 (0.15, 0.68)
40-44	3.59 (1.15, 11.17)	0.07 (0.05, 0.10)	0.14 (0.07, 0.30)
Age (10 years)	1.03 (0.89, 1.18)	0.68 (0.65, 0.72)	1.76 (1.61, 1.91)
Age by eGFR			
20-24	1.01 (0.86, 1.18)	1.02 (0.96, 1.09)	1.02 (0.92, 1.14)
25-29	0.93 (0.80, 1.08)	1.06 (0.99, 1.13)	1.03 (0.94, 1.14)
30-34	0.80 (0.69, 0.93)	1.25 (1.19, 1.33)	1.03 (0.94, 1.13)
35-39	0.82 (0.71, 0.95)	1.30 (1.23, 1.38)	1.09 (0.99, 1.19)
40-44	0.85 (0.73, 0.98)	1.28 (1.21, 1.35)	1.18 (1.07, 1.29)
Male vs female	0.85 (0.82, 0.89)	1.09 (1.05, 1.13)	1.22 (1.18, 1.26)
Number of eGFR tests[c]	1.05 (1.04, 1.05)	1.03 (1.03, 1.04)	1.06 (1.05, 1.06)
Diabetes	1.03 (0.99, 1.07)	1.48 (1.42, 1.54)	1.15 (1.12, 1.19)
Myocardial infarction	0.99 (0.93, 1.06)	0.93 (0.88, 1.00)	1.19 (1.13, 1.24)
Congestive heart failure	1.31 (1.25, 1.37)	1.25 (1.20, 1.31)	1.96 (1.89, 2.02)
Stroke/TIA	1.12 (1.07, 1.17)	1.01 (0.96, 1.06)	1.24 (1.20, 1.29)
Peripheral vascular disease	1.11 (1.03, 1.20)	1.09 (1.01, 1.18)	1.50 (1.42, 1.58)
ACEi/ARB	1.04 (1.00, 1.09)	1.12 (1.07, 1.18)	0.77 (0.74, 0.80)
NSAIDs	1.30 (1.25, 1.36)	0.93 (0.88, 0.97)	0.89 (0.86, 0.93)
Statin	0.97 (0.93, 1.01)	1.01 (0.97, 1.05)	0.79 (0.76, 0.82)

Model log-likelihood (LL) -116443.7 (model without interaction between age and eGFR, LL -116471.1)

Progr. or KF: Progression or kidney failure
[a]Categories of albuminuria based on converted and unconverted ACR (albumin-creatinine-ratio) defined as A1 (<3 mg/mmol), A2 (3-29 mg/mmol), A3<60 (30-59 mg/mmol), and A3≥60 (≥60 mg/mmol). Converted values were obtained using validated conversion method. Conversion factor for ACR from mg/mmol to mg/g: 0.113.
[b]Categories of index eGFR in 5 ml/min/1.73 m^2 increments
[c]Number of eGFR measurements during the qualifying period pefore cohort entry)
ACEi, angiotensin-converting enzyme inhibitor; ARB, angiotensin-receptor blocker; eGFR, estimated glomerular filtration rate (in mL/min/1.73 m^2); KF: kidney failure; NSAIDs, non-steroidal anti-inflammatory drugs; TIA, transient ischemic attack.

© 2022 Pasternak M et al. *JAMA Network Open.*

eTable 4. Cause-Specific Hazard Ratios for Outcomes (Interaction Model)

	**Hazard ratio (95% confidence interval)**		
**Covariates**	**Regression**	**Progr. or KF**	**Death**
Albuminuria[a]			
A1	1 (reference)	1 (reference)	1 (reference)
A2	0.47 (0.32, 0.70)	2.01 (1.43, 2.81)	0.92 (0.77, 1.11)
$A3_{<60}$	0.36 (0.13, 1.01)	3.33 (2.11, 5.23)	0.90 (0.55, 1.46)
$A3_{\geq60}$	0.17 (0.09, 0.35)	7.34 (5.33, 10.11)	1.00 (0.77, 1.30)
Index eGFR[b]			
15-19	1 (reference)	1 (reference)	1 (reference)
20-24	1.31 (0.33, 5.24)	0.58 (0.31, 1.10)	0.50 (0.19, 1.29)
25-29	2.03 (0.54, 7.71)	0.26 (0.14, 0.49)	0.32 (0.13, 0.80)
30-34	3.34 (0.90, 12.35)	0.24 (0.14, 0.41)	0.31 (0.13, 0.73)
35-39	2.93 (0.81, 10.58)	0.15 (0.09, 0.25)	0.16 (0.07, 0.38)
40-44	1.65 (0.46, 5.97)	0.08 (0.05, 0.14)	0.07 (0.03, 0.15)
Albuminuria by eGFR			
A2, 20-24	1.17 (0.76, 1.79)	0.98 (0.65, 1.49)	1.28 (1.03, 1.60)
$A3_{<60}$, 20-24	0.84 (0.27, 2.57)	0.74 (0.42, 1.29)	1.07 (0.61, 1.87)
$A3_{\geq60}$, 20-24	1.05 (0.48, 2.32)	0.86 (0.57, 1.27)	1.35 (0.98, 1.85)
A2, 25-29	1.24 (0.83, 1.87)	1.10 (0.74, 1.65)	1.27 (1.03, 1.56)
$A3_{<60}$, 25-29	0.93 (0.31, 2.81)	1.11 (0.64, 1.93)	1.30 (0.76, 2.23)
$A3_{\geq60}$, 25-29	1.23 (0.57, 2.64)	0.83 (0.56, 1.23)	1.57 (1.16, 2.12)
A2, 30-34	1.49 (1.00, 2.23)	0.88 (0.62, 1.26)	1.39 (1.14, 1.70)
$A3_{<60}$, 30-34	1.11 (0.36, 3.38)	0.87 (0.53, 1.42)	1.74 (1.01, 3.01)
$A3_{\geq60}$, 30-34	1.20 (0.55, 2.59)	0.74 (0.53, 1.03)	1.34 (0.99, 1.82)
A2, 35-39	1.61 (1.09, 2.39)	0.85 (0.60, 1.20)	1.42 (1.17, 1.73)
$A3_{<60}$, 35-39	1.26 (0.43, 3.66)	0.78 (0.48, 1.26)	1.76 (1.04, 2.98)
$A3_{\geq60}$, 35-39	1.69 (0.81, 3.52)	0.68 (0.49, 0.94)	1.48 (1.10, 1.99)
A2, 40-44	1.86 (1.26, 2.75)	0.98 (0.69, 1.39)	1.59 (1.31, 1.93)
$A3_{<60}$, 40-44	1.83 (0.64, 5.25)	1.06 (0.65, 1.71)	1.69 (1.00, 2.88)
$A3_{\geq60}$, 40-44	2.05 (0.98, 4.29)	0.85 (0.61, 1.18)	1.74 (1.29, 2.34)
Age (10 years)	0.97 (0.83, 1.13)	0.70 (0.66, 0.73)	1.67 (1.52, 1.83)
Age by eGFR			
20-24	1.02 (0.87, 1.21)	1.01 (0.94, 1.08)	1.06 (0.95, 1.18)
25-29	0.96 (0.81, 1.13)	1.04 (0.97, 1.12)	1.09 (0.98, 1.21)
30-34	0.84 (0.72, 0.99)	1.23 (1.16, 1.30)	1.08 (0.98, 1.20)
35-39	0.87 (0.75, 1.02)	1.26 (1.19, 1.34)	1.15 (1.04, 1.26)
40-44	0.91 (0.78, 1.06)	1.27 (1.20, 1.35)	1.25 (1.13, 1.38)
Male vs female	0.85 (0.82, 0.89)	1.09 (1.05, 1.13)	1.22 (1.18, 1.26)
Number of eGFR tests[c]	1.05 (1.04, 1.06)	1.03 (1.03, 1.04)	1.06 (1.05, 1.06)
Diabetes	1.02 (0.98, 1.07)	1.48 (1.42, 1.54)	1.15 (1.11, 1.19)
Myocardial infarction	0.99 (0.93, 1.06)	0.93 (0.88, 1.00)	1.19 (1.13, 1.25)
Congestive heart failure	1.31 (1.25, 1.37)	1.25 (1.20, 1.31)	1.95 (1.89, 2.02)
Stroke/TIA	1.12 (1.07, 1.17)	1.01 (0.96, 1.06)	1.24 (1.20, 1.28)
Peripheral vascular disease	1.11 (1.03, 1.20)	1.09 (1.01, 1.18)	1.50 (1.42, 1.58)
ACEi/ARB	1.04 (0.99, 1.09)	1.12 (1.07, 1.18)	0.77 (0.74, 0.79)
NSAIDs	1.30 (1.25, 1.36)	0.93 (0.88, 0.97)	0.89 (0.86, 0.93)
Statin	0.97 (0.93, 1.01)	1.01 (0.97, 1.05)	0.79 (0.76, 0.82)

Model log-likelihood (LL) -116410.3 (Likelihood ratio test (chisq.) vs model without albuminuria by eGFR interaction (eTable 2) 66.8 [DF 15], p<0.01)

Progr. or KF: Progression or kidney failure

© 2022 Pasternak M et al. *JAMA Network Open.*

[a]Categories of albuminuria based on converted and unconverted ACR (albumin-creatinine-ratio) defined as A1 (<3 mg/mmol), A2 (3-29 mg/mmol), A3<60 (30-59 mg/mmol), and A3≥60 (≥60 mg/mmol). Converted values were obtained using validated conversion method. Conversion factor for ACR from mg/mmol to mg/g: 0.113.
[b]Categories of index eGFR in 5 ml/min/1.73 m^2 increments
[c]Number of eGFR measurements during the qualifying period pefore cohort entry)
ACEi, angiotensin-converting enzyme inhibitor; ARB, angiotensin-receptor blocker; eGFR, estimated glomerular filtration rate (in mL/min/1.73 m^2); KF: kidney failure; NSAIDs, non-steroidal anti-inflammatory drugs; TIA, transient ischemic attack.

© 2022 Pasternak M et al. *JAMA Network Open.*

eTable 5. Cause-Specific Hazard Ratios for Regression (Sensitivity and Subgroup Analyses) Associated With Albuminuria

Model#	Albuminuria category	Hazard ratio	2.5% confidence limit	97.5% confidence limit
Main analysis (N=58,004)	A1	1 (reference)	-	-
	A2	0.75	0.72	0.79
	A3a	0.47	0.4	0.54
	A3b	0.27	0.24	0.3
Sensitivity analysis (N=58,004)	A1	1 (reference)	-	-
	A2	0.7	0.66	0.74
	A3a	0.41	0.34	0.49
	A3b	0.22	0.19	0.25
Albuminuria type ACR (N=27,052)	A1	1 (reference)	-	-
	A2	0.71	0.67	0.76
	A3a	0.45	0.38	0.54
	A3b	0.21	0.18	0.25
Albuminuria type DIP/PCR (N=30,952)	A1	1 (reference)	-	-
	A2	0.8	0.75	0.86
	A3a	0.47	0.34	0.64
	A3b	0.38	0.32	0.46
Albuminuria 0-1 year before entry (N=42,444)	A1	1 (reference)	-	-
	A2	0.73	0.7	0.78
	A3a	0.46	0.39	0.55
	A3b	0.27	0.24	0.31
Albuminuria 1-3 years before entry (N=15,560)	A1	1 (reference)	-	-
	A2	0.79	0.72	0.86
	A3a	0.5	0.35	0.7
	A3b	0.25	0.18	0.35
Prior outpatient eGFR(N=56,131)	A1	1 (reference)	-	-
	A2	0.76	0.73	0.8
	A3a	0.47	0.4	0.55
	A3b	0.27	0.24	0.3
No prior outpatient eGFR(N=2,641)	A1	1 (reference)	-	-
	A2	0.49	0.35	0.7
	A3a	0.47	0.19	1.19
	A3b	0.26	0.12	0.58
Sustained albuminuria (N=14,346)	A1	1 (reference)	-	-
	A2	0.69	0.65	0.73
	A3a	0.42	0.35	0.5
	A3b	0.24	0.21	0.27
Non-sustained albuminuria (N=43,658)	A1	1 (reference)	-	-
	A2	0.8	0.76	0.85
	A3a	0.64	0.47	0.87
	A3b	0.39	0.3	0.5

#Models are non-interaction models including the same covariates as in eTable 3.

© 2022 Pasternak M et al. *JAMA Network Open.*

eTable 6. Cause-Specific Hazard Ratios, Subdistribution Hazard Ratios for Regression Associated With Albuminuria

Model	eGFR[a]	Albuminuria category	Hazard ratio[b]	2.5% confidence limit	97.5% confidence limit
Non-interaction Cause-specific Cox regression	15-44	A1	1 (reference)		
		A2	0.75	0.72	0.79
		A3a	0.47	0.4	0.54
		A3b	0.27	0.24	0.3
Non-interaction Sub-distribution Fine and Gray regression	15-44	A1	1 (reference)		
		A2	0.67	0.64	0.7
		A3a	0.39	0.33	0.45
		A3b	0.16	0.15	0.19
Interaction Cause-specific Cox regression	40-44	A1	1 (reference)		
		A2	0.88	0.82	0.95
		A3a	0.66	0.51	0.86
		A3b	0.36	0.28	0.45
	35-39	A1	1 (reference)		
		A2	0.77	0.71	0.83
		A3a	0.46	0.34	0.62
		A3b	0.29	0.24	0.37
	30-34	A1	1 (reference)		
		A2	0.71	0.63	0.8
		A3a	0.4	0.26	0.63
		A3b	0.21	0.15	0.29
	25-29	A1	1 (reference)		
		A2	0.59	0.52	0.67
		A3a	0.34	0.22	0.51
		A3b	0.21	0.16	0.29
	20-24	A1	1 (reference)		
		A2	0.55	0.46	0.66
		A3a	0.3	0.19	0.48
		A3b	0.18	0.13	0.26
	15-19	A1	1 (reference)		
		A2	0.47	0.32	0.7
		A3a	0.36	0.13	1.01
		A3b	0.17	0.09	0.35
Interaction Sub-distribution Fine and Gray regression	40-44	A1	1 (reference)		
		A2	0.78	0.72	0.84
		A3a	0.55	0.43	0.71
		A3b	0.24	0.19	0.3
	35-39	A1	1 (reference)		

© 2022 Pasternak M et al. *JAMA Network Open.*

		A2	0.68	0.63	0.73
		A3a	0.35	0.26	0.47
		A3b	0.18	0.14	0.23
	30-34	A1	1 (reference)		
		A2	0.61	0.54	0.68
		A3a	0.29	0.19	0.45
		A3b	0.11	0.08	0.15
	25-29	A1	1 (reference)		
		A2	0.55	0.48	0.62
		A3a	0.31	0.2	0.47
		A3b	0.15	0.11	0.21
	20-24	A1	1 (reference)		
		A2	0.5	0.42	0.59
		A3a	0.3	0.19	0.48
		A3b	0.11	0.08	0.16
	15-19	A1	1 (reference)		
		A2	0.43	0.3	0.63
		A3a	0.29	0.1	0.83
		A3b	0.08	0.04	0.18

Legend: Non-interaction models do not include the interaction between albuminuria and eGFR; interaction models do.
Models are adjusted for the same variables included in the Cox models summarized in eTables 2-3.
eGFR[a] indicates the index eGFR category in 5 ml/min/1.73 m^2 increments from 15 to 44.
Hazard ratio[b] indicates the exponentiated coefficient of the model, cause-specific hazard ratio for Cox regression and sub-distribution hazard ratio for the Fine and Gray model.

© 2022 Pasternak M et al. *JAMA Network Open.*

eFigure 1. Derivation of Study Cohort

**Number of residents registered in Alberta Health Apr 1, 1994 – Mar 31, 2017 (N)**	**4,983,956**	
**Common exclusion criteria (N excluded):**	**4,644,966**	
• No serum creatinine measurements, May 1, 2002 – Mar 31, 2017	1,573,084	
• No outpatient serum creatinine measurements	201,969	
• All outpatient serum creatinine were tested under 18 years old	31,971	
• All outpatient serum creatinine measurements were after out-migration, registration end or accrual end (March 31, 2017)	6,099	
• Never had an outpatient eGFR measurement <60 ml/min/1.73 m^2	2,697,464	
• Only 1 outpatient eGFR <60 ml/min/1.73 m^2	129,412	
• The first eGFR less than 60 was already <15 ml/min/1.73 m^2	4,967	
**Incident CKD stage (N)**	**G3b (54,725)**	**G4 (18,674)**
**Unique exclusion criteria (N excluded):**	**284,265**	**320,316**
• Did not meet the sustained eGFR criterion for specific stage, for >90 days	246,755	307,004
• Index date was not between Apr 1, 2008 and Mar 31, 2017	36,510	12,197
• Index date was on the earliest date of death, out-migration, registration end or accrual end	20	8
• At least 1 outpatient eGFR <15 ml/min/1.73 m^2 prior to the qualifying period	282	541
• Initiated kidney replacement therapy on or prior to index date	698	566
**G3b-G4 CKD Cohort**	**65,509**	
No proteinuria measurements in the 3 years look-back window	7,505	
G3b-G4 CKD with at least 1 proteinuria measurements within 3 years of meeting G3b G4 CKD criteria (**Cohort size N**)	**58,004**	

Legend

- Alberta population registry started in 1994. Serum creatinine database started on May 1, 2002, with nearly complete coverage from July 1, 2003 (~98% of the Alberta population) and complete coverage from January 1, 2005 (100%). The ~1/3 of the population (n=1,573,084) who never had an eGFR measurement largely comprises people who were in the registry before the study start date (April 1, 2009). We considered all the available information preceding Apr 1, 2008 (look-back window for eGFR measurements and other data) to minimize the inclusion of prevalent cases in the incident CKD cohorts between Apr 1, 2008, and Mar 31, 2017.

© 2022 Pasternak M et al. *JAMA Network Open.*

Of those who were registered for at least 1 fiscal year between Apr 1, 2008, and Mar 31, 2017, 22% did not have a serum creatinine measurement. Most of these (~85%) were younger than 40 years, an expected figure considering that a screening test for serum creatinine is not recommended for most people under 40 in the absence of risk factors for CKD.

- Sustained eGFR criterion for specific stage: eGFR 30-44 ml/min/1.73 m^2 for G3b CKD and 15-29 ml/min/1.73 m^2 for G4 CKD.
- G3b-G4 CKD cohort: if an individual appeared in both G3b and G4 CKD cohorts, only the observation in G3b cohort was used (first encounter).

© 2022 Pasternak M et al. *JAMA Network Open.*

eFigure 2. Study Participants With Missing Albuminuria Measures Per Year Prior to the Study Period

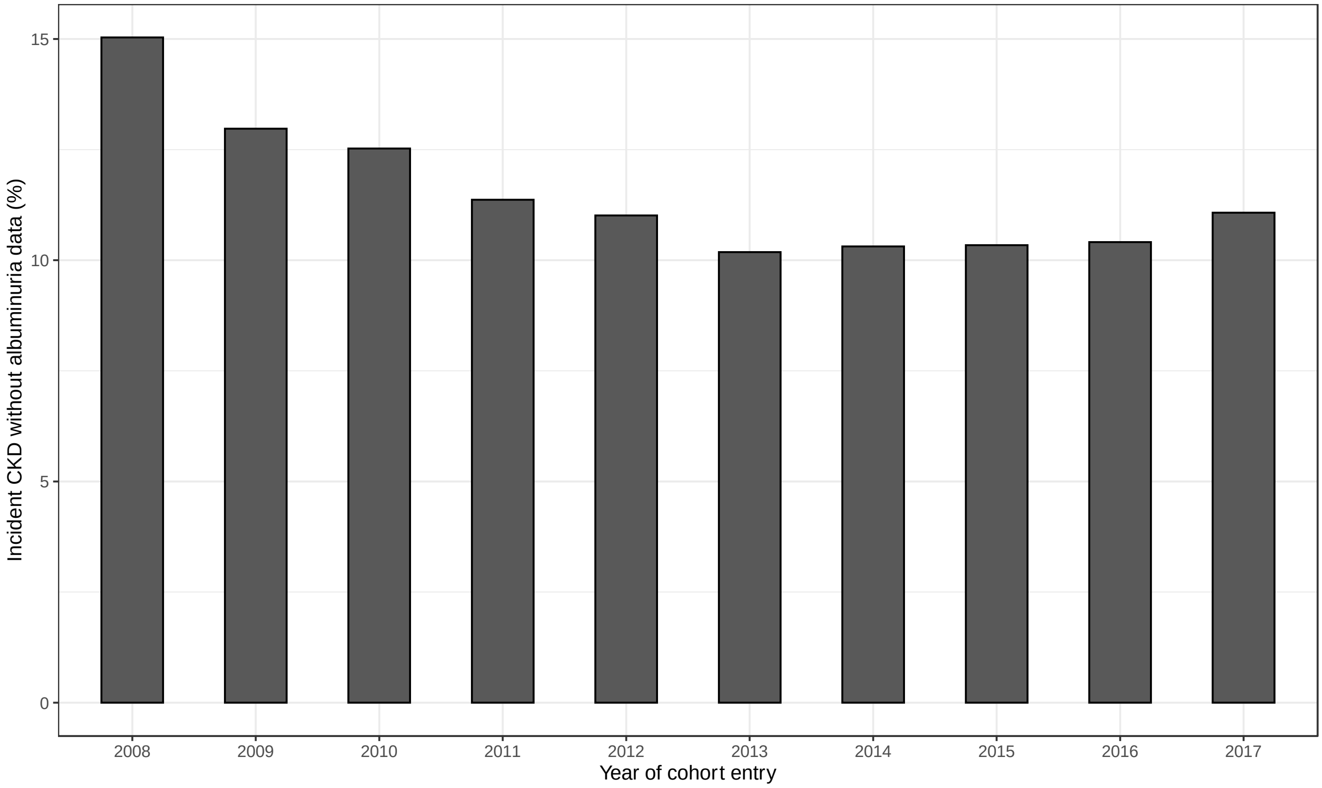

Bars refer to the percentage of incident adults with CKD who were not tested for albuminuria during the study accrual period.

© 2022 Pasternak M et al. *JAMA Network Open.*

eFigure 3. Cumulative Incidence Functions for Outcomes in People With Complete and Missing Albuminuria Data

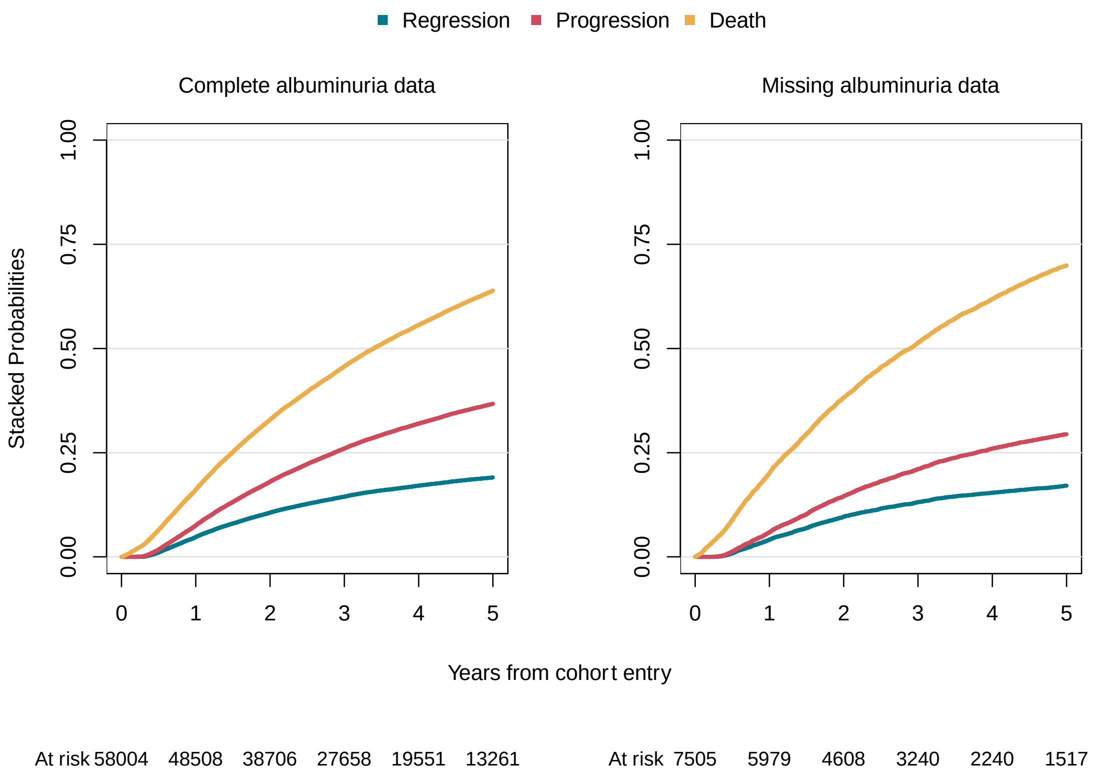

Legend: Cumulative incidence of CKD regression, CKD progression (progression or kidney failure), and death without regression, progression or kidney failure for participants with and without available measures of albuminuria.

© 2022 Pasternak M et al. *JAMA Network Open.*

eFigure 4. Association Between eGFR and CKD Regression Across Albuminuria Categories

	No. of Events	HR	95% CI	p-value
**Model 2**				
A1, eGFR 40-44	3313	1.65	[0.46-5.97]	=0.44
A1, eGFR 35-39	2566	2.93	[0.81-10.58]	=0.10
A1, eGFR 30-34	1129	3.34	[0.9-12.35]	=0.07
A1, eGFR 25-29	771	2.03	[0.54-7.71]	=0.30
A1, eGFR 20-24	382	1.31	[0.33-5.24]	=0.70
A1, eGFR 15-19 (ref)	82	1		
A2, eGFR 40-44	915	3.07	[0.91-10.38]	=0.07
A2, eGFR 35-39	773	4.72	[1.39-15.98]	=0.01
A2, eGFR 30-34	365	4.98	[1.44-17.25]	=0.01
A2, eGFR 25-29	322	2.53	[0.71-8.96]	=0.15
A2, eGFR 20-24	185	1.53	[0.41-5.72]	=0.53
A2, eGFR 15-19 (ref)	41	1		
A3<60, eGFR 40-44	61	3.03	[0.7-13.16]	=0.14
A3<60, eGFR 35-39	43	3.68	[0.84-16.16]	=0.08
A3<60, eGFR 30-34	20	3.7	[0.81-17.01]	=0.09
A3<60, eGFR 25-29	23	1.89	[0.41-8.81]	=0.42
A3<60, eGFR 20-24	20	1.1	[0.23-5.34]	=0.91
A3<60, eGFR 15-19 (ref)	4	1		
A3≥60, eGFR 40-44	75	3.39	[1.01-11.42]	=0.05
A3≥60, eGFR 35-39	84	4.94	[1.47-16.64]	=0.01
A3≥60, eGFR 30-34	20	4.01	[1.15-13.94]	=0.03
A3≥60, eGFR 25-29	23	2.5	[0.71-8.83]	=0.15
A3≥60, eGFR 20-24	20	1.37	[0.37-5.13]	=0.64
A3≥60, eGFR 15-19 (ref)	10	1		
A1 (ref)	8243	1		
A2	2601	0.47	[0.32-0.7]	<0.01
A3<60	171	0.36	[0.13-1.01]	=0.05
A3≥60	287	0.17	[0.09-0.35]	<0.01

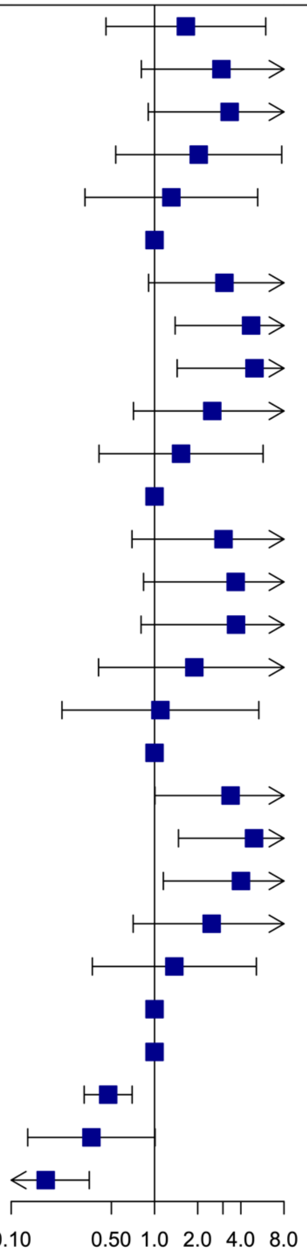

Model 2 is the same model as Model 2 in Figure 4. Model 2 includes the same covariates as Model 1 in Figure 4 (eTable 3), with the additional interaction between albuminuria and index eGFR category (eTable 4). This model shows the linear combinations of the coefficients (epidemiological formulation) instead of differences in log-hazard ratios (statistical interaction formulation) to summarize the association between eGFR and CKD regression across categories of albuminuria.

© 2022 Pasternak M et al. *JAMA Network Open.*

eFigure 5. Crude 5-Year Risk of Outcomes by Category of Albuminuria (Sensitivity Analysis)

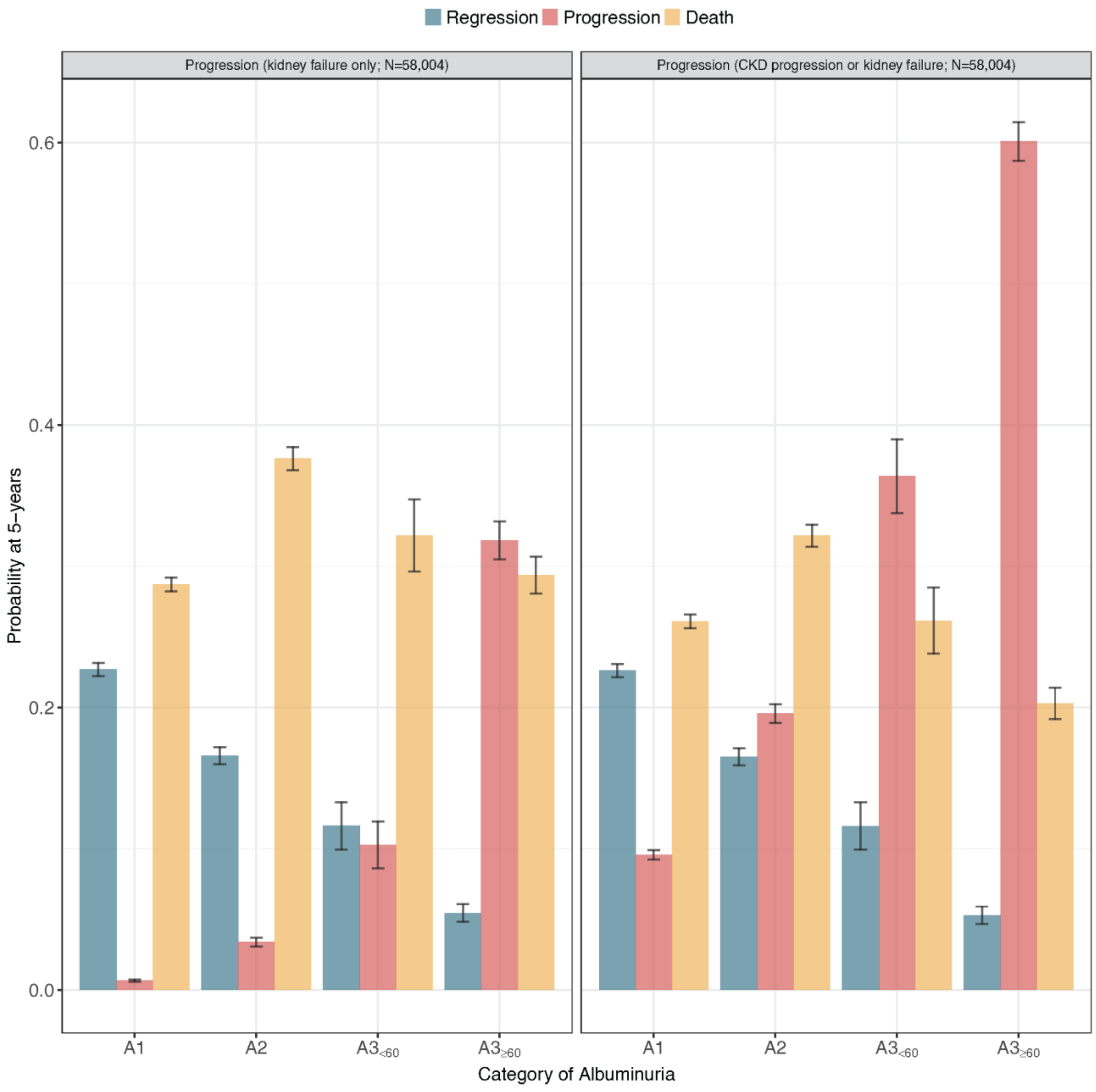

Outcome probabilities were estimated using cumulative incidence functions at 5 years after study entry by category of albuminuria. Progression represents chronic kidney disease (CKD) kidney failure only (i.e., sustained eGFR <10 ml/min/1.73 m2 or initiation of chronic kidney replacement therapy). Death refers to death without regression or kidney failure.

© 2022 Pasternak M et al. *JAMA Network Open.*

eFigure 6. Crude 5-Year Risk of Outcomes by Diabetes Status

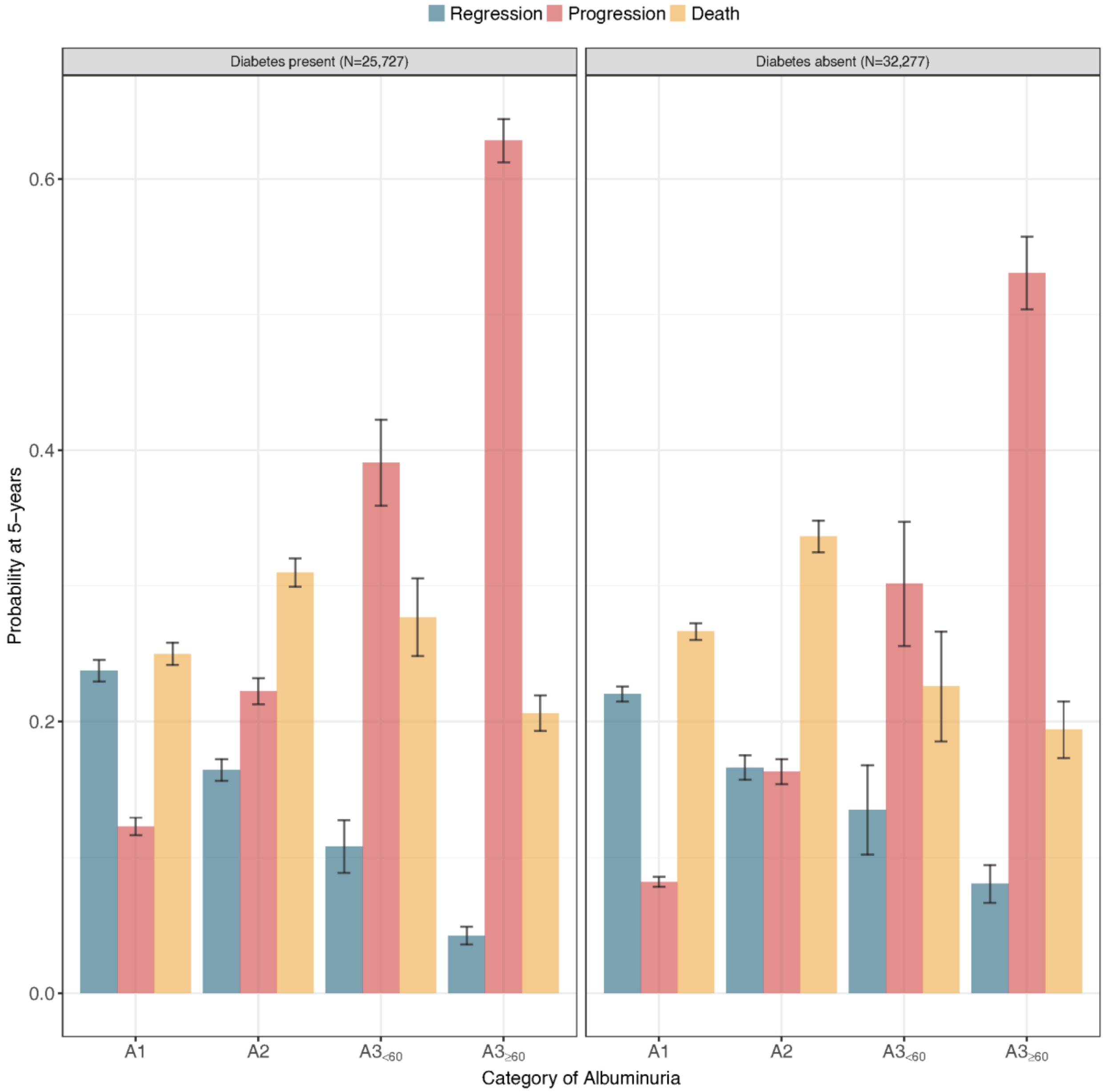

Outcome probabilities were estimated using cumulative incidence functions at 5 years after study entry by category of albuminuria. Progression represents chronic kidney disease (CKD) progression or kidney failure. Death refers to death without regression, progression or kidney failure.

© 2022 Pasternak M et al. *JAMA Network Open.*

eFigure 7. Crude 5-Year Risk of Outcomes According to the Type of Albuminuria Measurement

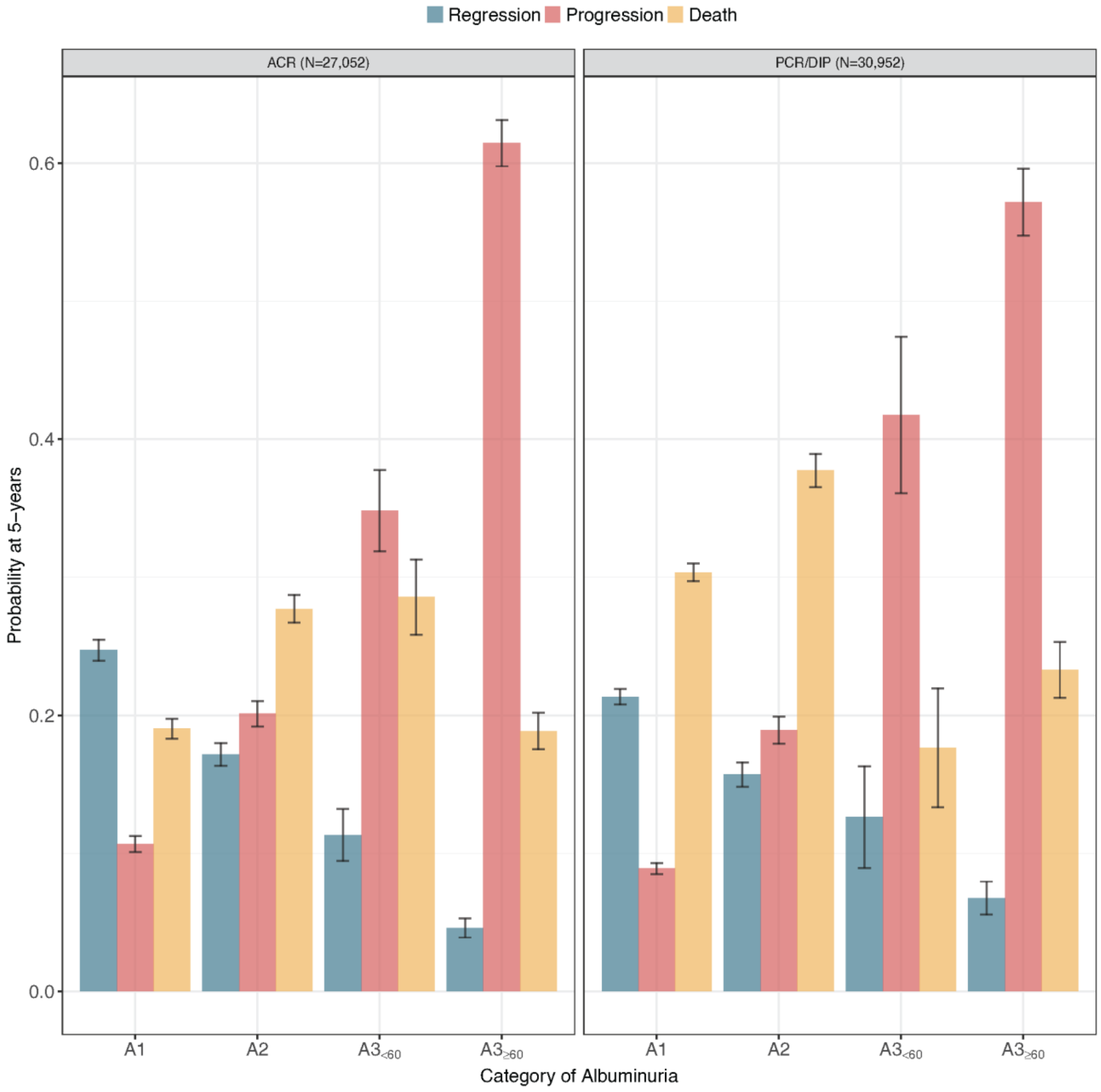

Outcome probabilities were estimated using cumulative incidence functions at 5 years after study entry by category of albuminuria. Progression represents chronic kidney disease (CKD) progression or kidney failure. Death refers to death without regression, progression or kidney failure. ACR: albumin to creatinine ratio; PCR: protein to creatinine ratio; DIP: dipstick urine protein.

© 2022 Pasternak M et al. *JAMA Network Open.*

eFigure 8. Crude 5-Year Risk of Outcomes According to Timing of Albuminuria Measurement

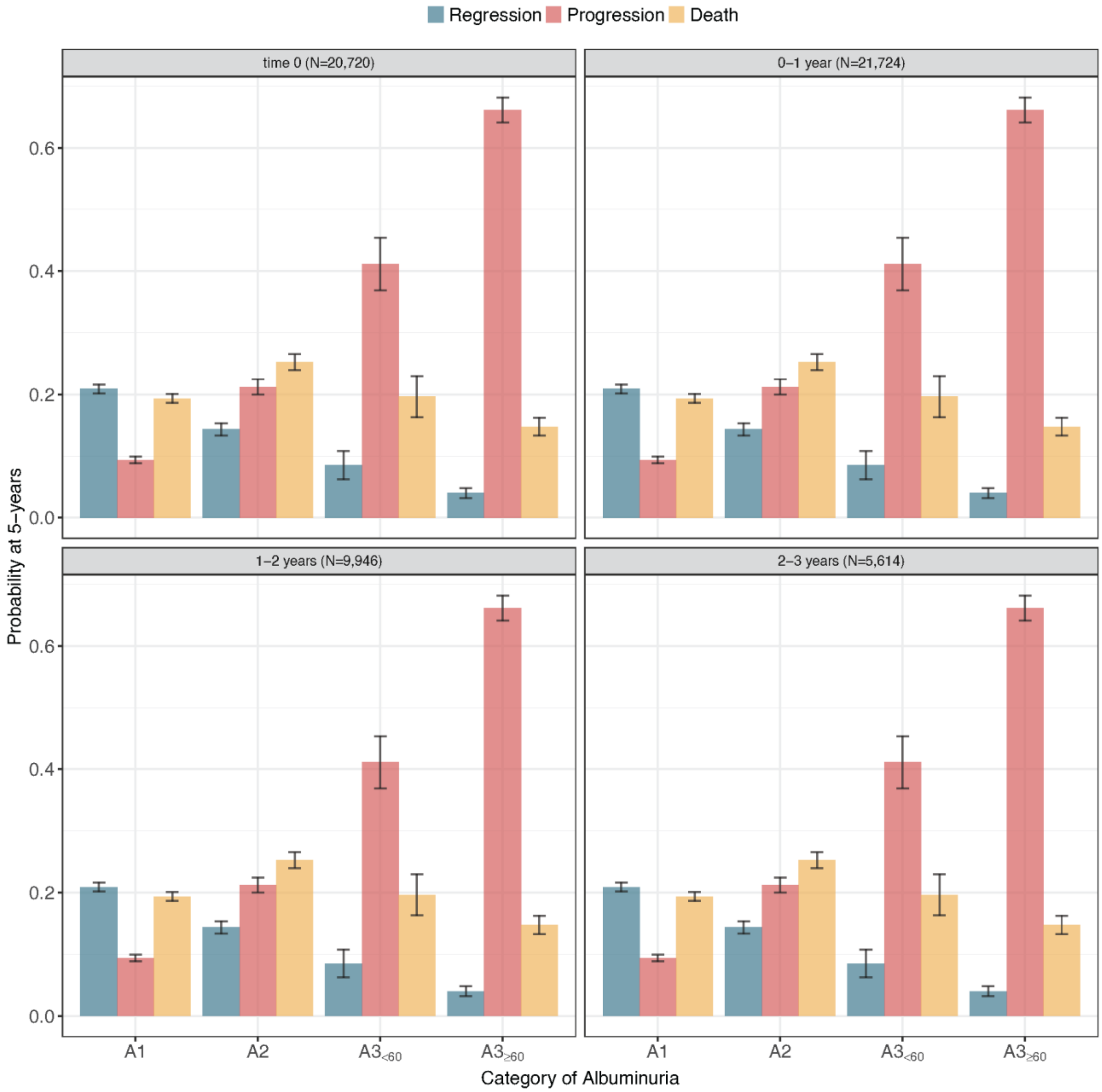

Outcome probabilities were estimated using cumulative incidence functions at 5 years after study entry by category of albuminuria. Progression represents chronic kidney disease (CKD) progression or kidney failure. Death refers to death without regression, progression or kidney failure. Time 0: albuminuria measured on the index date; time 0-1: albuminuria measured in the year before index date; time 1-2: albuminuria measured in the second year before index date; time 2-3: albuminuria measured in the third year before index date.

© 2022 Pasternak M et al. *JAMA Network Open.*

eFigure 9. Crude 5-Year Risk of Outcomes in Participants Without and With eGFR Before Study Entry

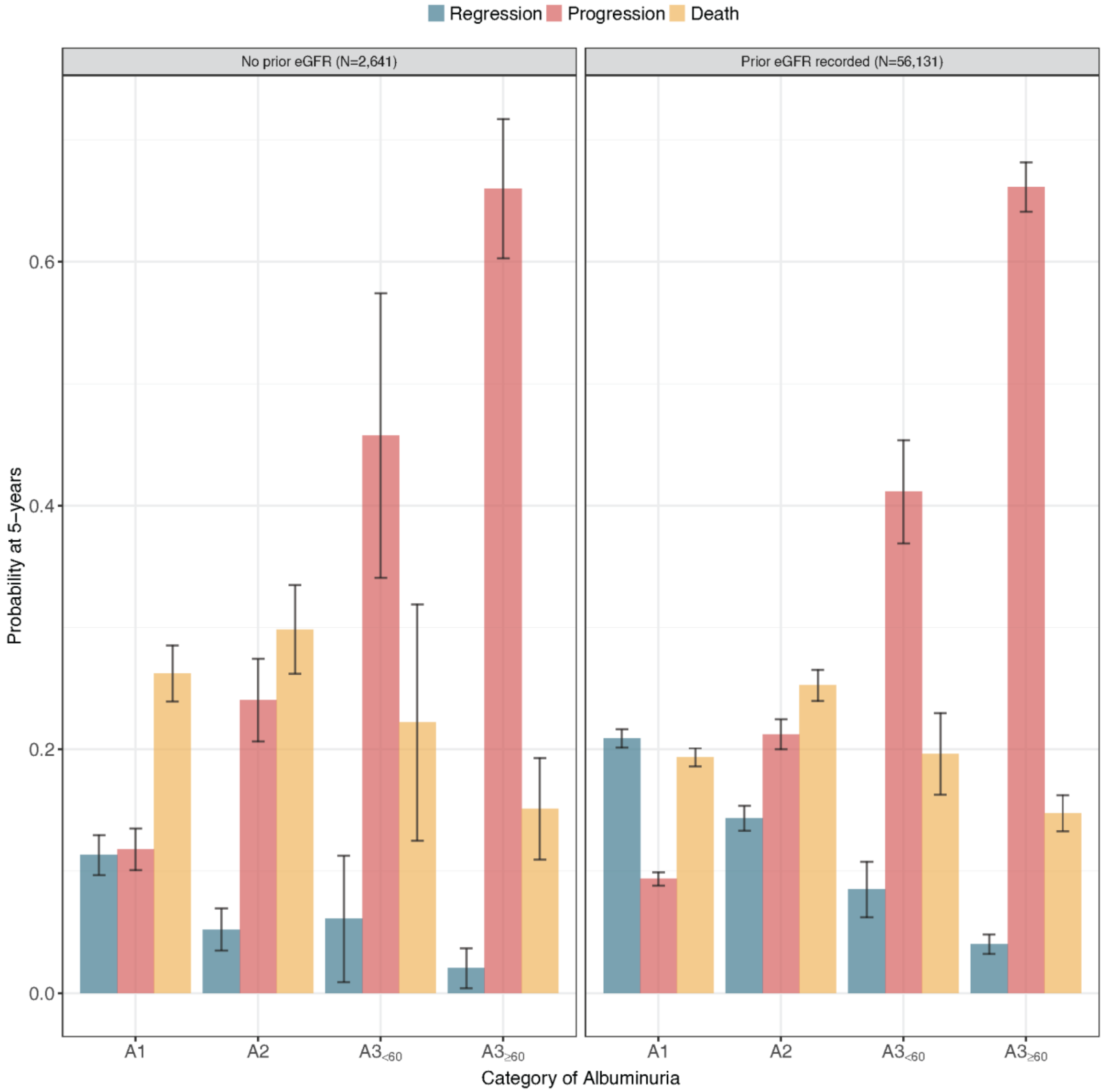

Outcome probabilities were estimated using cumulative incidence functions at 5 years after study entry by category of albuminuria. Progression represents chronic kidney disease (CKD) progression or kidney failure. Death refers to death without regression, progression or kidney failure.

© 2022 Pasternak M et al. *JAMA Network Open.*

eFigure 10. Crude 5-Year Risk of Outcomes in Participants With and Without Sustained Albuminuria at Baseline

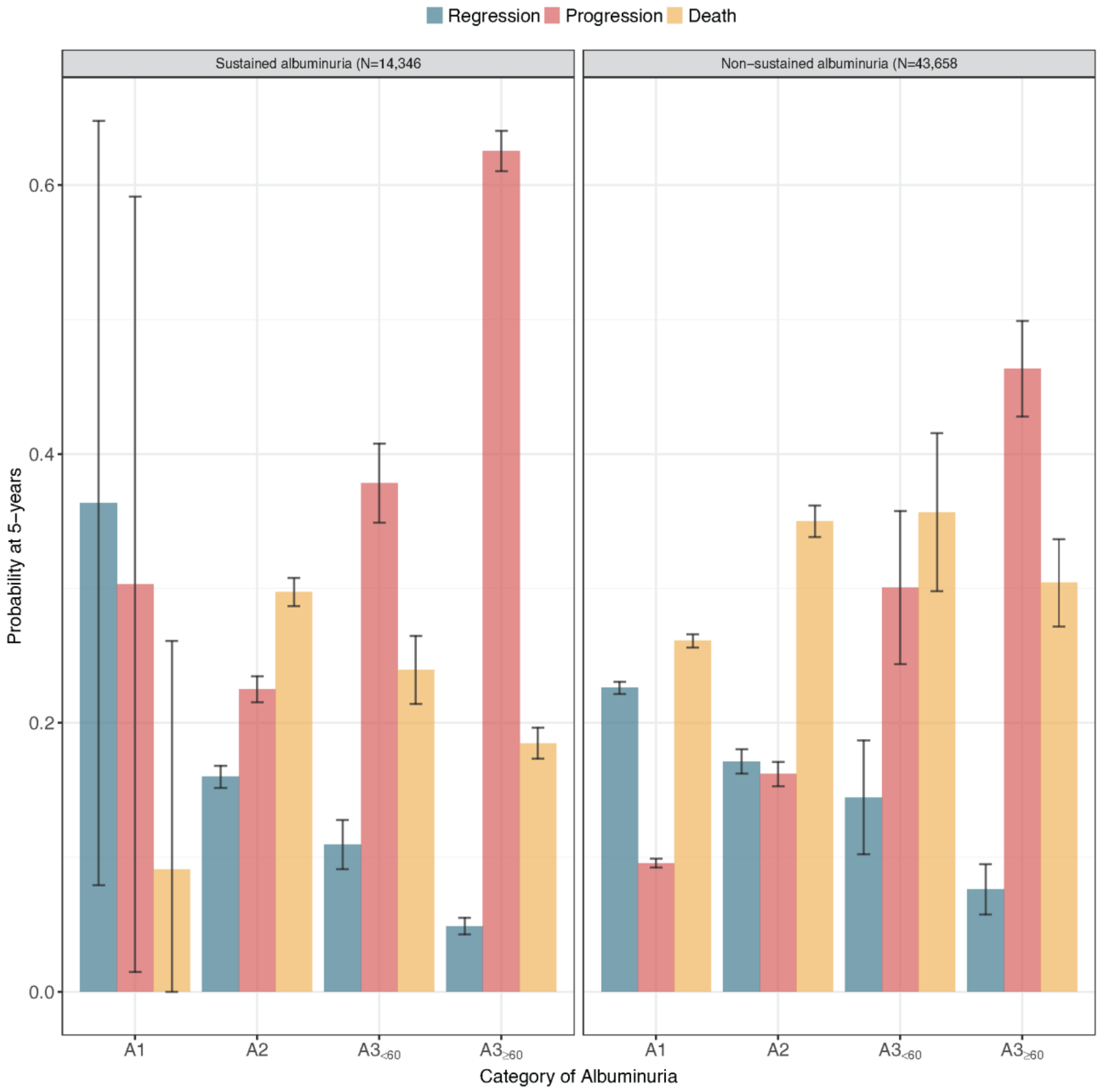

Outcome probabilities were estimated using cumulative incidence functions at 5 years after study entry by category of albuminuria. Progression represents chronic kidney disease (CKD) progression or kidney failure. Death refers to death without regression, progression or kidney failure.

© 2022 Pasternak M et al. *JAMA Network Open.*
